# The Last New Medicine

**Published:** 1858-01

**Authors:** 


*The last new Medicine.*—There is a new medicine coming out, which is destined to eclipse all the hair balms and purgatorial purgatives. The name has not been absolutely fixed upon, but a majority of the proprietors are inclined to call it the Odontoanneneakaton, or Compound Extract of Coral and Caoutchouc. You are Greek scholar enough to recognize, at once, its use and value. The observation having been made that children are much assisted at the time of dentition by biting India-rubber rings and coral, an acute gentlemen thought that a combination of the two would have perhaps a decided effect in restoring the teeth of elderly females. From this hint the medicine has been brought forth. The dentists, of course, are down upon it, but with a certificate from some chemist of the undoubted purity of the extract, and with the patronage of the Female Medical College, the proprietors may defy the whole profession. When it comes into general use you shall be duly informed.—*Med. & Surg. Reporter.*

